# Complement Dysregulation in Infancy: A Case of CD59 Deficiency With Neurological Sequelae

**DOI:** 10.7759/cureus.84085

**Published:** 2025-05-14

**Authors:** Fadi Busaleh, Heeba Y Al Kalaf, Nabil Almajhad, Raneem Alhalal, Jana M Alqahtani, Zahra Almuslem, Nouf AlQurashi, Manar A Alhejji, Zainab I Albeladi, Sarah B Alessa

**Affiliations:** 1 Pediatric, Maternity and Children's Hospital, Al-Ahsa, SAU; 2 Pediatric Neurology, Maternity and Children's Hospital, Al-Ahsa, SAU; 3 Division of Neurology, Pediatric Department, Maternity and Children's Hospital, Al-Ahsa, SAU; 4 Medicine, Vision College, Riyadh, SAU; 5 College of Medicine, Vision College, Riyadh, SAU; 6 Medicine, College of Medicine, King Saud Bin Abdulaziz University for Health Sciences, Riyadh, SAU; 7 College of Medicine, King Faisal University, Al-Ahsa, SAU

**Keywords:** acute flaccid paralysis, cd59 deficiency, immune hemolytic anemia, infantile hypotonia, infantile stroke, non-immune hemolytic anemia, paroxysmal nocturnal hemolytic anemia

## Abstract

CD59 deficiency is a rare autosomal recessive disorder causing complement-mediated hemolysis, strokes, and neuropathy. Early recognition is critical to avoid irreversible complications. We report a two-year-old girl, born to consanguineous parents, who presented with recurrent hypotonia, motor regression, ischemic stroke, and Coombs-negative hemolytic anemia. Whole exome sequencing confirmed a homozygous CD59 mutation (c.323C>A; p.Ser108). She was treated with immune-moderating therapy and anticoagulants with a rehabilitation program. Despite therapy, she developed persistent neurological deficits and failure to thrive. CD59 deficiency should be considered in infants with unexplained hypotonia, stroke, and hemolytic anemia. Early diagnosis and complement inhibition may reduce complications.

## Introduction

The human immune system consists of various cells and pathways that work together to defend against pathogens. It is broadly classified into acquired and innate immunity [1].

Innate immunity serves as the first line of defense, responding rapidly to threats through diverse mechanisms. These include physical and chemical barriers such as the skin, immune effector cells like granulocytes and natural killer (NK) cells, antimicrobial peptides that directly target pathogens, and soluble mediators such as cytokines, including tumor necrosis factor-alpha (TNF-α) and interleukin-6 (IL-6), and serum proteins like the complement system [2].

The complement system plays a critical role in host defense by activating a series of signaling proteins that bind to harmful microbes. This binding triggers a cascade of events that culminate in the formation of the membrane attack complex (MAC), which creates pores in microbial cell membranes, leading to cell lysis and the elimination of pathogens. To prevent damage to host cells, this process is tightly regulated by key proteins such as the glycosyl phosphatidylinositol (GPI)-anchored (CD59). CD59 functions as a "suicide inhibitor," halting the activation cascade and protecting host cells from complement-mediated damage. This regulation is essential for maintaining immune homeostasis and preventing unintended harm to the body’s own cells [3,4].

CD59 deficiency is a rare autosomal recessive disorder characterized by recurrent hemolytic anemia, ischemic strokes, and peripheral neuropathy. Mutations in the CD59 gene impair the function of the CD59 protein, a key regulator of the complement system, leading to uncontrolled complement activation and subsequent clinical manifestations. Early recognition and treatment are crucial to prevent irreversible complications [5]. We are discussing a case of CD59 deficiency as the first case encountered in Saudi Arabia and the challenges encountered.

## Case presentation

A two-year-old girl, born to consanguineous parents with an unremarkable prenatal and perinatal history, first presented at seven months with generalized hypotonia following a febrile illness. She exhibited motor regression, losing abilities such as sitting with support and rolling over. Initial evaluations, including sepsis workup, cerebrospinal fluid analysis, and metabolic screening, were unremarkable. Neurological assessment noted generalized hypotonia, absent reflexes, and tongue fasciculations, with normal consciousness. Spinal muscular atrophy was considered, but genetic testing was negative. Despite rehabilitation efforts, she experienced recurrent episodes of hypotonia, each associated with febrile illnesses, leading to cumulative motor deficits.

**Figure 1:**
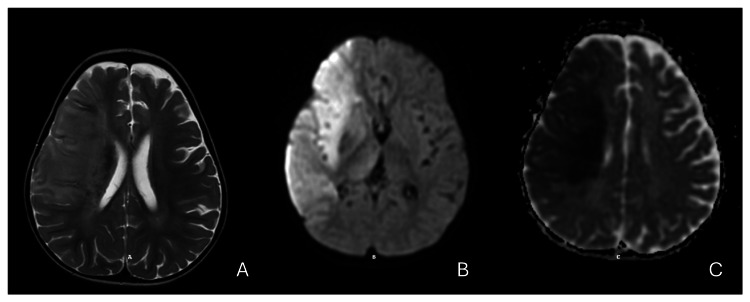
A) T2-weighted imaging: it shows a hyperintense signal seen in the deep white matter following the distribution of the right middle cerebral artery with brain edema and midline shift. B) DWI (diffusion-weighted imaging): showing a bright hyperintensity (restricted diffusion) noted in the right deep white matter area, and C) ADC (apparent diffusion coefficient) map showed a corresponding low signal (hypointensity) in the same region, confirming true restricted diffusion. This confirms acute ischemic infarction involving the right-sided temporoparietal peritoneal middle cerebral artery.

At 18 months, she presented with acute left-sided weakness and decreased consciousness, without preceding fever. Neuroimaging confirmed a right middle cerebral artery ischemic stroke; this will be demonstrated in the brain magnetic resonance (MRI) images (Figure 1). Concurrent Coombs-negative hemolytic anemia was noted, with hemoglobin dropping to 7 g/dL, necessitating red blood cell transfusion. Subsequently, she required admission to the pediatric intensive care unit (PICU), where she was intubated and placed on mechanical ventilation for respiratory support. Anticoagulation therapy was initiated with low molecular weight heparin to prevent further coagulopathy, and later transitioned to aspirin.

Given the combination of neurological events and hemolytic anemia, whole exome sequencing was performed, revealing a homozygous mutation in the CD59 gene (NM_203330.2:c.323C>A; p․Ser108), confirming the diagnosis of CD59 deficiency. Eculizumab, a complement inhibitor, was initiated as a disease-modifying therapy.

At the latest assessment, she was alert, with a tracheostomy and nasogastric feeding tube in place. She exhibited failure to thrive (head circumference below the third percentile) and left-sided hemiparesis. Generalized weakness was more pronounced on the left, with absent reflexes and bilateral equinovarus deformity.

## Discussion

The complement membrane attack complex (MAC) is a pore-forming protein complex on the cell surface that leads to osmotic lysis of targeted cells or organisms. This process is utilized by both innate and adaptive immunity. Regulation of MAC activity is mediated by various complement regulators, including CD46, CD55, and CD59. Specifically, CD59 controls the final steps of MAC formation by binding between C8 and C9, thereby preventing pore formation. Dysregulation of this process results in uncontrolled MAC activity, leading to cell death and unrestrained osmotic lysis [6-8].

CD59 deficiency can lead to diverse clinical presentations depending on the affected body system. For example, the deficiency may cause chronic hemolytic anemia and is linked to the paroxysmal nocturnal hemoglobinuria condition characterized by intravascular hemolysis, hemoglobinuria, and vascular thrombosis [5,9,10].

Conversely, neurological manifestations of CD59 deficiency may cause recurrent hypotonia due to demyelination, initially presenting as acute inflammatory demyelinating polyradiculoneuropathy (AIDP) and potentially progressing to chronic inflammatory demyelinating polyneuropathy (CIDP). Additionally, patients are at risk for acute cerebrovascular events, such as stroke [5,11].

The patient initially presented in infancy with hypotonia and was evaluated for spinal muscular atrophy, the most recognized cause. Genetic testing revealed a heterozygous duplication with three copies of the SMN1 gene for exons 7 and 8. Although this novel mutation initially raised concerns, further segregation analysis showed that the mother also carried the gene but was healthy, indicating that it is a variant of uncertain significance. Given the broad differential for hypotonia, including neuromuscular, metabolic, mitochondrial, and neuro-immunological causes, the diagnostic approach was aligned with both local and international studies [12,13].

After the patient developed an acute ischemic stroke, likely resulting from a hypercoagulable state, and subsequent acute non-immune-mediated hemolytic anemia, alternative differential diagnoses were considered. Repeat genetic testing revealed a CD59 gene defect, a homozygous mutation in exon 6 (NM_203330.2:c.323C>A, p․Ser108). This confirmed CD59 deficiency, with the phenotype matching the genotype, which is the typical diagnostic pathway for such cases. These clinical presentations were similar to those reported in several studies, particularly by Almutawea et al., the largest study in the Arabian Gulf region, which observed that infants initially present with acute flaccid paralysis, followed by later manifestations of pure hematological involvement or cerebrovascular events [14].

The patient was started on eculizumab, an immune-modulating therapy that targets complement protein C5. By binding to C5, eculizumab prevents its cleavage into C5a and C5b, thereby halting the formation of the membrane attack complex (MAC) early in the complement cascade. This inhibition of MAC formation reduces complement-mediated cell lysis. These effects are consistent with findings from both local and international literature, including studies by Legendre et al., Almutawea et al., Rother et al., and Hillmen et al., which demonstrated that eculizumab is effective in reducing hemolytic events and thrombotic complications [14-17].

On the other hand, the patient was started on aspirin as a symptomatic measure to help prevent further cerebrovascular events and was enrolled in a comprehensive rehabilitation program. Even with this multidisciplinary treatment plan, the patient continued to experience tragic outcomes. Literature, including studies by Wassmer et al. and Manguinao et al., suggests that the severity of clinical outcomes is directly related to the degree of demyelination [18,19]. As observed in our patient, the particularly frequent demyelinating episodes likely contributed to the unfavorable prognosis.

## Conclusions

This case highlights the critical need for a broad differential diagnosis in infants presenting with hypotonia and recurrent neurological deficits. Although initial evaluations focused on more common neuromuscular disorders, comprehensive genetic testing ultimately identified CD59 deficiency. Despite targeted therapy with eculizumab and symptomatic treatment with aspirin, persistent demyelinating episodes adversely affected the outcome. These findings emphasize the importance of early, thorough diagnostic assessments to ensure appropriate management in complex cases.

## References

[1] Ardicli D, Taskiran EZ, Kosukcu C (2017). Neonatal-onset recurrent Guillain-Barré syndrome-like disease: clues for inherited CD59 deficiency. Neuropediatrics.

[2] Medzhitov R, Janeway C Jr (2000). Innate immune recognition: mechanisms and pathways. Immunol Rev.

[3] Merle NS, Church SE, Fremeaux-Bacchi V, Roumenina LT (2015). Complement system part I - molecular mechanisms of activation and regulation. Front Immunol.

[4] Kim DD, Song WC (2006). Membrane complement regulatory proteins. Clin Immunol.

[5] Nevo Y, Ben-Zeev B, Tabib A (2013). CD59 deficiency is associated with chronic hemolysis and childhood relapsing immune-mediated polyneuropathy. Blood.

[6] Ricklin D, Hajishengallis G, Yang K, Lambris JD (2010). Complement: a key system for immune surveillance and homeostasis. Nat Immunol.

[7] Höchsmann B, Schrezenmeier H (2015). Congenital CD59 deficiency. Hematol Oncol Clin North Am.

[8] Farkas I, Baranyi L, Ishikawa Y (2002). CD59 blocks not only the insertion of C9 into MAC but inhibits ion channel formation by homologous C5b-8 as well as C5b-9. J Physiol.

[9] Motoyama N, Okada N, Yamashina M, Okada H (1992). Paroxysmal nocturnal hemoglobinuria due to hereditary nucleotide deletion in the HRF20 (CD59) gene. Eur J Immunol.

[10] Yamashina M, Ueda E, Kinoshita T (1990). Inherited complete deficiency of 20-kilodalton homologous restriction factor (CD59) as a cause of paroxysmal nocturnal hemoglobinuria. N Engl J Med.

[11] Ben-Zeev B, Tabib A, Nissenkorn A (2015). Devastating recurrent brain ischemic infarctions and retinal disease in pediatric patients with CD59 deficiency. Eur J Paediatr Neurol.

[12] Leyenaar J, Camfield P, Camfield C (2005). A schematic approach to hypotonia in infancy. Paediatr Child Health.

[13] Jan M (2007). The hypotonic infant: Clinical approach. Jr Ped Neur.

[14] Almutawea LM, Hajeri AA, Farid EM (2023). Inherited CD59 deficiency, where neurology and genetics intertwine. Neurosciences (Riyadh).

[15] Legendre CM, Licht C, Muus P (2013). Terminal complement inhibitor eculizumab in atypical hemolytic-uremic syndrome. N Engl J Med.

[16] Rother RP, Rollins SA, Mojcik CF (2007). Discovery and development of the complement inhibitor eculizumab for the treatment of paroxysmal nocturnal hemoglobinuria. Nat Biotechnol.

[17] Hillmen P, Hall C, Marsh JC (2004). Effect of eculizumab on hemolysis and transfusion requirements in patients with paroxysmal nocturnal hemoglobinuria. N Engl J Med.

[18] Wassmer E, Billaud C, Absoud M (2024). Long term outcome in non-multiple sclerosis paediatric acquired demyelinating syndromes. Eur J Paediatr Neurol.

[19] Manguinao M, Krysko KM, Maddike S (2019). A retrospective cohort study of plasma exchange in central nervous system demyelinating events in children. Mult Scler Relat Disord.

